# The Phosphorus Compounds of the Cell Nucleus

**Published:** 1952-06

**Authors:** W. M. McIndoe, J. N. Davidson

## Abstract

**Images:**


### THE PHOSPHORUS COMPOUNDS OF THE CELL NUCLEUS.

W. M. McINDOE AND J. N. DAVIDSON.

*From the Department of Biochemistry, The University of Glasgow.*

Received for publication April 15, 1952.

THE three chief chemical constituents of the cell nucleus are deoxyribonucleic acid (DNA), a basic protein (histone or protamine), and a non-histone protein containing tryptophan (Stedman and Stedman, 1943, 1947, 1951 ; Mirsky and Pollister, 1946). It is now generally agreed that the cell nucleus also contains small amounts of ribonucleic acid (RNA) (Brachet, 1942 ; Davidson and Waymouth, 1944*a* ; Euler and Hahn, 1946 ; Vendrely and Vendrely, 1948 ; Bergstrand, Eliasson, Hammarsten, Norberg, Reichard and Ubisch, 1948), which has been reported both in the nucleolus (Caspersson and Schultz, 1940 ; Brachet, 1942 ; Davidson and Waymouth, 1946 ; Pollister and Leuchtenberger, 1949*a* ; Lagerstedt, 1948) and in the chromosomes (Mirsky and Ris, 1947).

In addition to DNA and RNA the nucleus also contains phospholipids and small amounts of other phosphorus compounds such as phosphopıoteins (Davidson, Gardner, Hutchison, McIndoe, Raymond and Shaw, 1949 ; Davidson and McIndoe, 1949). In this paper such compounds are studied with the aid of radioactive phosphorus ($^{32}$P).

### METHODS.

### (a) *Isolation of nuclei.*

Calf thymus glands obtained from the slaughter-house as soon as possible after the death of the animal were homogenized in citric acid in a Waring blender or M.S.E. Nelco blender running at half speed, with mixing vessel surrounded by an ice jacket. The entire isolation procedure was carried out in the cold room or in a refrigerated centrifuge using the technique described by Mirsky and Pollister (1946), and was controlled in the later stages by frequent microscopic examinations.

The same procedure was used to obtain rabbit, rat and fowl liver nuclei (Fig. 1). The animals were killed by cervical dislocation or by exsanguination under ether anaesthesia and the livers immediately perfused with 0·9 per cent sodium chloride solution before being removed, chilled and homogenized. Nuclei were also prepared from the GRCH 15 tumour originally described by Peacock (1933).

For the preparation of fowl erythrocyte nuclei the method of Dounce and Lan (1943) was used.

In all cases the suspensions of clean nuclei were dried from the frozen state after small measured portions had been taken for dilution and counting in a haemocytometer. The dry weight of a given number of nuclei could thus be obtained. Photomicrographs of the isolated rat liver nuclei are shown in Fig. 1.

(b) *Radioactive phosphorus administration.*

$^{32}P$ obtained from the Atomic Energy Research Establishment, Harwell, was administered as carrier-free inorganic phosphate by subcutaneous or intramuscular injection in doses of 100–500μc. per kg. body weight 2 or 4 hours before the animals were killed.

(c) *Analytical methods.*

(i) *Schmidt-Thannhauser* (1945) *technique* (*Fig.* 2).—Phosphorus compounds were separated by the method of Schmidt and Thannhauser (1945) as modified by Davidson, Frazer and Hutchison (1951). A weighed portion of dried nuclei

Fig. 2.—Scheme of separation of phosphorus compounds in nuclei (modified from Schmidt and Thannhauser, 1945).

was extracted three times with 10 per cent trichloracetic acid (TCA). The extracts were combined (acid soluble 1), but little attention was paid to this fraction since clearly much of the soluble P must already have been removed during the isolation of the nuclei. The extracted residue was treated with successive portions of acetone, ethanol, ethanol-chloroform (3:1), warm ethanol-ether (3:1) and ether. These extracts were taken to dryness and the residue taken up in chloroform to form the phospholipid fraction.

The dry nucleoprotein residue was incubated overnight at 37° with N NaOH to hydrolyse RNA to acid soluble nucleotides. A small amount of material failed to dissolve in warm alkali and was centrifuged down and washed with small amounts of N NaOH (Insoluble 1). The alkaline digest and washings were then neutralised with 5N HCl and treated with 0·5 vol. 30 per cent TCA. The precipitate of protein and DNA, after standing for 30 minutes in ice, was centrifuged down and washed twice with 10 per cent TCA. This precipitate (fraction

" DNAP ") was dissolved in 0·1 N NaOH while the acid supernatant fluid and washings were combined as fraction acid soluble 2.

A portion of this fraction was then treated with Mathison's (1909) reagent and ammonium hydroxide in order to precipitate inorganic phosphate (fraction " $P_2$ " of Davidson, Frazer and Hutchison (1951), derived from " phosphoprotein "). The phosphorus which remained in solution was treated as organic phosphate (fraction " organic P ").

Fractions " acid soluble 2 " and " organic P " should contain phosphorus derived from RNA in the form of acid soluble nucleotides (RNAP). Pentose was therefore estimated in " acid soluble 2 " by the orcinol method of Mejbaum

FIG. 3.—Combined Schneider and Schmidt-Thannhauser procedure.

(1939) and by the phloroglucinol method of Euler and Hahn (1946), using calibration curves made from a purified sample of yeast RNA, so that pentose could be read off directly in terms of ribonucleic acid phosphorus (RNAP).

Fraction " DNAP " should contain phosphorus derived from DNA only. Deoxypentose was therefore estimated by the diphenylamine method (Dische, 1930) as employed by Davidson and Waymouth (1944*b*), using a calibration curve prepared from a purified specimen of thymus DNA so that readings could be made directly in terms of deoxyribonucleic acid phosphorus (DNAP).

Phosphorus estimations were made on all samples by the method of Allen (1940).

The optical densities of fractions " acid soluble 2 " and " DNAP " were read in a Beckman Model DU spectrophotometer at 260 mμ. and 290 mμ. The difference in the readings was proportional to the amount of nucleic acid present as determined from standard solutions of RNA and DNA treated in the same way. The samples to be examined were diluted to contain less than 4 $\mu$g. nucleic acid P/ml. and were read against a reagent blank.

The radioactivity of each fraction was determined on a Type M6 liquid counter (20th Century Electronics) attached to a conventional scaling unit.

(ii) *The Schneider* (1945) *technique.*—A weighed portion of dried nuclei was extracted with TCA and lipid solvents, as described above. The extracted material was treated with 5 per cent TCA at 90° for 15 minutes to split off nucleic acids as acid soluble hydrolysis products. The residue was washed twice with 5 per cent TCA and was then dissolved in N NaOH to form the " Schneider residue". The combined TCA extract and washings formed the " Schneider extract". Phosphorus, pentose and deoxypentose were determined on both extract and residue, and ultraviolet absorption measurements made on the extract.

(iii) *Combined Schneider-Schmidt-Thannhauser procedure* (*Fig.* 3).—It was apparent at an early stage that although the " Schneider residue " might have been expected to contain only phosphoprotein P, its phosphorus content was in fact very much greater than that of fraction $P_2$. The " Schneider " residue clearly contained a mixture of phosphorus compounds, and it was therefore submitted to a Schmidt-Thannhauser separation by incubating in alkaline solution at 37° for 18 hours. An insoluble residue (" Insoluble 2 ") was centrifuged down and washed with dilute alkali. The alkaline digest with washings was then acidified with HCl and TCA as described above and separated into a precipitate (" ester phosphate residue ") and an acid extract (" acid soluble 3 ").

Inorganic phosphate ($P_3$) was precipitated from the latter leaving an organic acid soluble phosphorus fraction (" ester phosphate "). Phosphorus and, where necessary, pentose and deoxypentose were determined in these fractions.

(iv) *Methods used in ionophoresis experiments.*—The separation of individual nucleotides from fraction " acid soluble 2 " was carried out by the method of ionophoresis on paper described by Smellie and Davidson (1951) and Davidson and Smellie (1952). In such experiments the alkaline digest was prepared with 0·3 N KOH and was subsequently acidified with 60 per cent perchloric acid. The " acid soluble 2 " fraction prepared in this way was brought to pH 3·5 by addition of alkali and dispensed on to a strip of Whatman 3 MM filter-paper (72 cm. × 7 cm.), which was moistened with 0·02 M citric acid-trisodium citrate buffer pH 3·5. To obtain adequate separation of the nucleotides the run was carried out at a potential gradient of about 10·7 volts/cm. for 18 hours. The ribonucleotides are preceded by components which run off the end of the paper under the conditions described above. To demonstrate them a separate run on a strip 57 cm. × 7 cm. was carried out at a potential gradient of 13·6 volts/cm. for 6 hours.

After a run the paper was dried and the bands located by the ultra-violet light method of Holiday and Johnson (1949) and marked lightly in pencil. Photographic records were made by the method of Markham and Smith (1949), and occasional autoradiographs were made by leaving the paper in contact with Kodak Industrex type D film for about 18 days.

The portions of the paper containing the bands were cut out and eluted according to Consden, Gordon and Martin (1947). The nature of each eluate was determined by its position on the paper and its ultraviolet absorption spectrum. Total phosphorus and radioactivity were also determined.

### RESULTS.

The results of a series of analyses are shown in Table I, in which the composition of bulk nuclei is given in terms of mg. P/100 g. dry weight. The phospholipid concentration is small, much below the value for whole tissue. The

TABLE I.—*Composition of Cell Nuclei from Various Sources and Specific Activities of Phosphorus in Liver Tissue 2 hours after administration of* $^{32}P$.

P. Amount of phosphorus in mg./100 g. dry weight of nuclei.
C. Specific activity in counts/min./100 $\mu$g. P.

| | Liver, rabbit. | | Liver, rat. | | Liver, rat (72 hr. fast). | | Thymus, calf. | Erythrocytes, fowl bearing GRCH tumour. |
|---|---|---|---|---|---|---|---|---|
| | P | C | P | C | P | C | P | P |
| Phospholid | 59 | 166 | 49 | 775 | 67 | 1640 | 30 | 101 |
| Alkali soluble 1 | 1890 | 145 | 1770 | 319 | 1710 | 449 | 2810 | 2440 |
| Acid soluble 2 : | | | | | | | | |
| Total P | 310 | 846 | 327 | 1980 | 315 | 2260 | 195 | 203 |
| RNAP by orcinol | 224 | — | 263 | — | 266 | — | 131 | 88 |
| by phloroglucinol | 216 | — | 265 | — | 242 | — | 108 | 90 |
| by UV absorption | — | — | 276 | — | 274 | — | 177 | — |
| Organic P | 275 | 702 | 297 | 1560 | 265 | 2010 | 161 | 191 |
| $P_2$ | 21 | 3380 | 14 | 3370 | 39 | 3430 | 23 | 5 |
| DNAP by phosphorus | 1610 | 16 | 1450 | 11 | 1410 | 15 | 2610 | 2240 |
| by diphenylamine | 1670 | — | 1320 | — | 1310 | — | 2480 | 2380 |
| by UV absorption | — | — | 1530 | — | 1380 | — | 2550 | — |
| *Schneider extract :* | | | | | | | | |
| Total P | 1430 | 124 | 1540 | 297 | 1400 | 373 | 2320 | 2070 |
| RNAP by orcinol | 193 | — | 238 | — | 249 | — | 111 | — |
| RNAP by phloroglucinol | 202 | — | 234 | — | 228 | — | 101 | 100 |
| DNAP by diphenylamine | 1540 | — | 1440 | — | 1370 | — | 2460 | 2440 |
| NAP by UV absorption | — | — | 1440 | — | 1370 | — | 2010 | — |
| *Schneider residue :* | 405 | 303 | 288 | 563 | 306 | 697 | 447 | 283 |
| Acid soluble 3 | 333 | 367 | 244 | 658 | 271 | 837 | 418 | — |
| $P_3$ | 19 | 3850 | 30 | 2410 | 45 | 2830 | 37 | — |
| Ester phosphate | 289 | 94 | 215 | 216 | 221 | 208 | 366 | — |
| Ester phosphate residue | 66 | 83 | 44 | 157 | 51 | 201 | 48 | — |

presence of appreciable amounts of phosphorus in fraction " acid soluble 2 " suggests that RNA is present in fairly large amounts in the cell nucleus, being more abundant in liver nuclei than in the nuclei of thymus or erythrocytes. Confirmation of the presence of RNA is given by the results of estimations of RNAP by the orcinol and phloroglucinol methods which show moderately good agreement, but figures obtained from measurements of ultraviolet absorption are rather more erratic. On the other hand the figure for " organic P " is frequently higher than that for RNAP. This is particularly evident in fowl erythrocyte nuclei, and may be due either to the presence of a variable quantity of an unknown phosphorus compound or to the leakage of a small amount of DNA into the " acid soluble 2 " fraction. The latter explanation holds for calf thymus nuclei, in which ultraviolet absorption measurements give a figure considerably higher than that obtained by pentose estimation, but in all types of nuclei subsequent experiments by ionophoresis on paper have conclusively proved the presence in the " acid soluble 2 " fraction of appreciable amounts of non-nucleotide phosphoric esters in addition to the expected nucleotides.

The difference between the figures for total P and " organic P " in the " acid soluble 2 " fraction is due to the presence of a small amount of inorganic phosphate (fraction $P_2$) which is derived from so-called " phosphoprotein." A similar fraction is present in whole tissue (Davidson, Frazer and Hutchison, 1951).

The amounts of DNAP estimated by the three different methods show moderately good agreement, although it is not unusual to find that, except in rabbit liver nuclei, the diphenylamine method tends to give a lower figure than do the other two methods.

Results obtained by the Schneider method are also shown in Table I. Although it might be expected that the Schneider extract would contain all the phosphorus derived from RNA and DNA, the figure obtained for this fraction is invariably

TABLE II.—*Mean Values for the Composition of the Single Cell Nucleus pg./nucleus.**

| | Thymus, calf. | Liver, rabbit. (Mean of 5.) | Liver, rat. (Mean of 5.) | Liver, rat (72 hr. fast). (Mean of 3.) | Liver, tumour-bearing fowl. | Erythrocyte, fowl. (Mean of 4.) | Erythrocyte, tumour-bearing fowl. (Mean of 3.) | GRCH 15 tumour, fowl. |
|---|---|---|---|---|---|---|---|---|
| Phospholipid | 0·21 | 0·70 | 2·14 | 0·93 | 0·19 | 0·48 | 0·40 | 10·3 |
| RNA by phosphorus estimation | 0·45 | 1·21 | 2·88 | 2·13 | 0·55 | 0·25 | 0·14 | 6·38 |
| by pentose estimation | 0·33 | 1·04 | 2·56 | 1·84 | 0·48 | 0·20 | 0·14 | 7·50 |
| by ultraviolet absorption | 0·48 | 1·05† | 2·68‡ | 1·89† | 0·51 | — | — | 6·32 |
| DNA by phosphorus estimation | 7·30 | 6·57 | 9·65 | 9·85 | 2·91 (2·57†) | 2·46 | 2·63 | 5·09 (4·90‡) |
| by diphenylamine reaction | 6·90 | 6·79 | 8·70 | 9·27 | 2·64 | 2·22 | 2·51 | 4·30 |
| by ultraviolet absorption | 7·13 | 6·24† | 9·69‡ | 9·76† | 2·74 | — | — | 5·68 |
| Nuclear mass | 28·2 | 44·4 | 79·4 | 66·5 | 16·5 | 11·1 | 9·9 | 217 |

* pg. (picogram) = $10^{-12}$ g.

† Mean of 2.

‡ Mean of 4.

lower than the sum of " organic P " and " DNAP". At the same time the Schneider residue, which might be expected to contain only phosphoprotein P, invariably shows a much higher phosphorus content than could be accounted for by fraction $P_2$. This discrepancy was observed by Schneider (1945, 1946) for whole tissue and has been commented on by Davidson, Frazer and Hutchison (1951). That the Schneider residue contains several different types of phosphorus compound is made abundantly clear when it is submitted to a Schmidt-Thannhauser fractionation (Table I). The protein-bound inorganic fraction " $P_3$ " obtained in this way corresponds roughly in amount to fraction " $P_2$". It therefore represents the true " phosphoprotein " phosphorus, and the other phosphorus compounds in the Schneider residue are almost certainly derived from the non-nucleotide esters mentioned above, and from nucleic acid phosphorus which has failed to be removed in the Schneider extraction although the portions of the nucleic acid molecules containing the reactive sugars have been split off from the protein. This is clear from the fact that, in general, the figures for RNAP obtained by pentose estimation on the Schneider extract agree with those obtained by pentose estimation on " acid soluble 2", while DNAP obtained by the diphenylamine method on the Schneider extract gives results which are comparable with those obtained from the Schmidt-Thannhauser DNAP fraction.

It is clear from Table I that dry preparations of calf thymus and fowl erythrocyte nuclei have a much higher DNA concentration and a lower RNA concentration than have liver nuclei, but a quite different light is shed on the results when they are expressed as amounts of each component per nucleus (Table II). The DNA content of the calf thymus nucleus (7·3 pg.) is of the same order as that found by Ris and Mirsky (1949), while the values for fowl nuclei (2·4 to 2·9 pg.) are very much lower and agree with the results of Davidson, Leslie, Smellie and Thomson (1950). The DNA content of nuclei from the GRCH 15 tumour of the fowl (5·1 pg.) is approximately double the value for normal fowl tissues. The figures for rat liver (9·65 pg.) are higher than those for other rat tissues, but are in agreement with the values found in a very extensive series of estimations at present being carried out in this department (Davidson, Heagy and Thomson, 1952, unpublished results).

TABLE III.—*Relative Composition of Nuclear Components Expressed in Moles P/10 Moles Adenylic P.*

| | Thymus, calf.* | Liver, rabbit. | Liver, rat.† | Liver, rat. | Liver, normal. cock. | Liver, tumour-bearing cock. | GRCH 15 tumour, cock. |
|---|---|---|---|---|---|---|---|
| Acid soluble 2 | 170·4 | 66·3 | 64·9 | 67·4 | 83·1 | 80·0 | 80·7 |
| Cytidylic acid | 10·7 | 13·1 | 14·3 | 12·1 | 14·6 | 17·7 | 15·4 |
| Adenylic acid | 10·0 | 10·0 | 10·0 | 10·0 | 10·0 | 10·0 | 10·0 |
| Component H | 8·4 | — | — | 2·2 | 4·5 | 2·6 | 1·7 |
| Guanylic acid | 22·9 | 13·6 | 14·8 | 13·4 | 27·0 | 22·3 | 28·7 |
| Uridylic acid | 19·0 | 14·0 | 12·9 | 11·7 | 14·4 | 13·9 | 14·3 |
| Component D | — | — | — | 3·3 | 4·1 | 4·1 | 4·2 |
| ,, C | — | — | — | | 3·4 | 3·6 | 6·8 |
| ,, B | 3·0 | — | — | 14·8 | 11·3 | 6·1 | 5·5 |
| ,, A | 4·3 | 7·3 | 7·2 | | | | |
| Inorganic fraction | 1·5 | 6·8 | — | 10·3 | 7·5 | 3·7 | 2·2 |
| Organic fraction | 2·8 | 0·5 | — | 4·5 | 3·8 | 2·4 | 3·3 |
| Purine/Pyrimidine ratio | 1·11 | 0·87 | 0·91 | 0·98 | 1·28 | 1·02 | 1·30 |

* The DNA derivative (see text) which lay between uridylic acid and component A and B accounted for 79·1 moles P in calf thymus nuclei.

† Mean of four preparations.

The RNA content of the individual nucleus is low in thymus and erythrocytes and is particularly high in the rat liver and in the GRCH 15 tumour. Nuclei from these last two sources have also a particularly high phospholipid content. The effect of a 72-hour fast is to reduce the amounts of phospholipid and RNA in rat liver nuclei and the nuclear mass, while the DNA content remains unchanged. The nuclear mass is particularly high in rat liver and in GRCH 15 tumour, but is low in the other fowl tissues.

It is clear from the results shown in Table I that fraction " acid soluble 2 " contains phosphorus compounds other than simple ribonucleotides and inorganic phosphate. In an attempt to separate these compounds from the nucleotides, and the ribonucleotides from each other, the method of ionophoresis on paper was employed with the results shown in Table III.

In all the nuclei examined the four ribonucleotides cytidylic acid, adenylic acid, guanylic acid, uridylic acid were identified by their absorption spectra. The order in which they occur on the paper is shown in Fig. 4, which is a photograph in ultraviolet light taken by the method of Markham and Smith (1949). In the material from one sample of rat liver nuclei each nucleotide was submitted to hydrolysis with perchloric acid (Marshak and Vogel, 1950), and the bases so liberated examined by paper chromatography by the method of Wyatt (1951). In each nucleotide only the expected base and no other was found. It is clear therefore that nuclear RNA is similar qualitatively to cytoplasmic RNA. We are indebted to Dr. G. Crosbie for the separation and estimation of the bases.

In different types of nuclei the proportions of the various nucleotides show only minor variations. Adenylic acid is least abundant and the purine to pyrimidine ratio is not from far unity. Using adenylic acid as a standard of reference (Table III) the most variable component is seen to be guanylic acid, which is particularly abundant in thymus nuclei, in the nuclei of the GRCH 15 tumour and in the liver nuclei of birds bearing this tumour. In most cases, particularly in thymus, a faint ultraviolet absorbing area was found on the paper between adenylic and guanylic acids (Fig. 4). The nature of this component, which we have termed H and which has properties very like those of adenylic acid itself, is as yet unknown, but it has been repeatedly detected in different kinds of nuclei.

In confirmation of the findings of Smellie and Davidson (1951) for whole liver tissue the nucleotides were found to be preceded by a fast-moving crescentic spot showing up faintly in an ultraviolet photograph (Fig. 5). This material, which we have designated component A, contains appreciable amounts of organic phosphate but inorganic phosphate is also present.

In early experiments with thymus nuclei and the nuclei of rabbit and rat liver component A alone was investigated, but subsequently, as the result of experiments with autoradiographs (see below), the whole area of the paper between component A and uridylic acid was examined by cutting it into sections corresponding to components B, C and D shown in Fig. 5. Component B lies immediately behind component A, from which it cannot readily be separated. It is probable that component B consists essentially of the inorganic moiety associated with component A. Component B should in fact correspond to fraction $P_2$ derived from phosphoprotein.

Component D lies immediately in front of uridylic acid and component C in the space between B and D ; both C and D are organic in nature. It is clear therefore that fraction " acid soluble 2 " contains in addition to the expected ribonucleotides at least four other components of which three are organic phosphates. These components are clearly visible in the autoradiographs shown in Fig. 4.

In material derived from calf thymus nuclei there was clear evidence that some breakdown of DNA had occurred with the liberation of fragments into the " acid soluble 2 " fraction, for a spot of free guanine was found at the starting-point while the ribonucleotides were preceded by a massive ultraviolet absorbing spot containing a large amount of phosphorus (7·91 mole.P/mole. adenylic acid P, Table III). This spot contained adenine together with equimolecular amounts of reactive deoxypentose and phosphorus, and has been provisionally identified as an adenine-guanine dinucleotide from which guanine has been split off.

The extent of incorporation of $^{32}P$ into the various phosphorus fractions of the cell nucleus during a 2-hour period is shown in Table I. The incorporation of $^{32}P$ into all fractions of fowl erythrocytes was so low as to make the results of little significance. The specific activity of the DNAP in rabbit and rat liver is very low indeed as compared with the cytoplasmic fractions, but by contrast the specific activity of the organic P fraction which contains ribonucleotides is very high indeed. In experiments in which we have compared nuclear RNAP and cytoplasmic RNAP the former has always shown a much higher rate of incorporation of $^{32}P$ than the latter (Davidson, McIndoe and Smellie, 1951). The specific activity of " organic P " is always less than that of fraction " acid soluble 2" from which it is derived. This is due to the presence in the latter of small amounts of " phosphoprotein " phosphorus ($P_2$), which has a very high activity indeed. As might be expected, the Schneider extract which contains breakdown products of RNA and DNA has a specific activity intermediate between that of "DNAP " and " organic P". The Schneider residue has an activity greater than that of the Schneider extract although much less than that of $P_2$, but when it is fractionated into its component parts, fraction $P_3$, which would appear to be true phosphoprotein phosphorus, corresponds both in amount and in activity to fraction $P_2$.

TABLE IV.—*Specific Activities (cts./min./100 μg. P) of Phosphorus in Nuclear Components. The nucleotides were obtained in a long run by ionophoresis on paper and the additional components in a short run.*

| | Liver, rat.* | Liver, rat. | Liver, normal cock. | Liver, tumour-bearing cock. | GRCH 15 tumour, cock. |
|---|---|---|---|---|---|
| Dosage of 32 P (μg./kg. of body weight) | 200 | 350 | 500 | 500 | 500 |
| Acid soluble 2 | 1,030 | 5,560 | 11,600 | 4,180 | 2,960 |
| Cytidylic acid | 833 | 4,370 | 10,200 | 3,150 | 2,040 |
| Adenylic acid | 1,000 | 5,210 | 11,800 | 3,490 | 2,150 |
| Component H | — | 2,200 | 10,800 | 2,940 | 1,330 |
| Guanylic acid | 641 | 4,180 | 9,510 | 2,690 | 1,910 |
| Uridylic acid | 837 | 4,150 | 11,400 | 3,920 | 2,580 |
| Component D | — | — | 7,970 | 3,350 | 2,010 |
| ,, C | — | — | 10,200 | 1,830 | 3,010 |
| ,, B | — | } 8,080 | } 16,340 | } 9,270 | } 5,640 |
| ,, A | 2,260 | | | | |
| Inorganic fraction of A + B | — | 10,910 | 27,600 | 14,000 | 13,000 |
| Organic fraction of A + B | — | 1,320 | 1,470 | 1,850 | 563 |
| DNAP | 10 | — | 346 | 1,270 | 1,340 |

* Mean of 4 with adenylic acid taken as 1000.

A more detailed picture of the specific activities of the components of fraction " acid soluble 2 " is given in Table IV, which shows the results of separation by ionophoresis on paper. The high specific activity of nuclear RNA is confirmed by isolation of the individual nucleotides. Although there is no great difference between the activity of the nucleotides, adenylic acid tends to show the highest activity. Components C and D have activities of the same order as those of the nucleotides, while the activities of components A and B together are considerably higher. When the combined components A and B are separated into organic and inorganic portions the inorganic fraction (which corresponds to $P_2$) is found to have a very high activity indeed, while the activity of the organic fraction is lower than that of the nucleotides. The general pattern obtained from GRCH 15 tumour is similar to that for other tissues.

The presence of components A, B, C and D is shown clearly in the autoradiograph in Fig. 4. The nucleotides themselves together with component H, which is moderately active, show up in the autograph in Fig. 5, in which the areas of radioactivity are seen to correspond closely with the ultraviolet absorbing areas in the adjacent ultraviolet print.

Since the " DNAP " fraction has a low specific activity and is precipitated from a solution of much greater specific activity, it is possible that this fraction may be contaminated with small amounts of phosphorus of high activity although it is washed several times. It was therefore thought necessary to investigate the effect of dissolving the " DNAP " fraction in alkali and reprecipitating with acid. One such reprecipitation reduced the activity by about 40 per cent, but further reprecipitations proved to be unnecessary and indeed undesirable since they tended to promote degradation of the material. In all experiments in which ionophoresis was employed one reprecipitation of the DNAP fraction was used.

From Table I it is clear that the specific activity of the DNAP in rabbit and rat liver is very low indeed, and this is again evident in Table IV. The DNAP of the liver of the normal cock on the other hand has a much higher specific activity, while that of the DNAP in the GRCH 15 tumour, as might be expected in a rapidly growing tissue, is still higher and approaches the value for the ribonucleotides (Table IV). It is of interest that the specific activity of the DNAP in the liver of the tumour-bearing bird is higher than that in the normal bird. This is in agreement with the observation of Kelly and Jones (1951) that the presence of a tumour in any part of the body causes an increased incorporation of $^{32}P$ in the DNAP of liver and spleen.

### DISCUSSION.

In examining results obtained from such a study as this consideration must be given to the degree of alteration which nuclei may undergo on isolation. Comparison of results obtained by chemical analysis and by the Feulgen staining technique applied to tissue sections suggests that no DNA is lost on isolation (Pollister and Leuchtenberger, 1949*b* ; Ris and Mirsky, 1949), and confirmation of this view is to be found in the close agreement between the results obtained by different workers using different methods for the mean DNA content of the single nucleus. On the other hand fairly convincing evidence for the loss of protein from the nucleus during the process of isolation from aqueous media has been reported by Pollister and Leuchtenberger (1949*b*) and by Dounce, Tishkoff, Barnett and Frier (1950), although this is contested by Stedman and Stedman (1951). To what extent loss of RNA and other phosphorus-containing constituents may occur during isolation in citric acid is quite unknown.

It is clear from the results shown in Table I that, as might be expected, the bulk of the phosphorus in the nucleus is present as DNA, but the results of the Schmidt and Thannhauser procedure reveal considerable amounts of phosphorus in the " acid soluble 2 " fraction. That a large proportion of this fraction consists of the ribonucleotides produced by alkaline hydrolysis of RNA is evident from the results of pentose estimations by the orcinol and phloroglucinol methods, and the results of the ionophoresis experiments have conclusively established the presence in nuclear RNA of the four nucleotides which are found in RNA

from other sources. The presence of RNA in the nucleus has been suspected from histochemical tests with enzymes (Brachet, 1942; Davidson and Waymouth, 1946), from the results of ultraviolet absorption measurements (Caspersson and Schultz, 1940), and as the result of positive pentose tests on isolated nuclei (Euler and Hahn, 1946; Vendrely and Vendrely, 1948; Villela, 1949; Nash, 1949).

The present work not only demonstrates the presence of RNA in the nucleus but indicates its composition, which is seen from Table III to vary from one type of nucleus to another, the relative amounts of guanylic acid being particularly high in material from calf thymus and the tumours and livers of the tumour-bearing fowl. In a preliminary note Elson and Chargaff (1951) quote figures for the composition of rat liver nuclear RNA which are somewhat similar to ours, although their proportions of guanylic and uridylic acids are rather lower than those shown here. Marshak (1951) quotes figures for the molar composition of calf thymus nuclear RNA in which the proportions of cytosine and uracil are very low indeed. If his ribonucleotide fraction were contaminated with a DNA derivative such as we have found in our calf thymus preparations such low figures might be expected.

Comparison of the results of the molar composition of nuclear RNA with cytoplasmic RNA from the same type of cell reveals wide differences. Both in rabbit and rat liver and in calf thymus the cytoplasmic RNA is characterised by a higher guanine content than is found in the corresponding nuclear RNA (Chargaff, 1950; Marshak, 1951; Davidson and Smellie, 1952). Guanine is particularly abundant in the nuclear RNA of the GRCH 15 tumour and of the fowl liver, but it is also present in high relative proportions in the cytoplasmic RNA from the same material (Beale, Harris and Roe, 1950).

These differences in composition between nuclear and cytoplasmic RNA together with the much more rapid incorporation of $^{32}P$ by the former make it quite clear that the RNA of the nucleus is a separate entity, and is not merely derived from cytoplasmic fragments adhering to the isolated nuclei. The presence of component H, however, which would appear to be a nucleotide of ionophoretic mobility intermediate between that of adenylic and guanylic acids, has not so far been detected in any cytoplasmic RNA fraction examined in this laboratory. Components A, B, C and D on the other hand appear in the ribo-

### EXPLANATION OF PLATES.

FIG. 1.—Isolated rat liver nuclei.

A. Photographed by phase contrast, magnification × 1600.

B and C. Photographed in ultraviolet light at 253·6 m$\mu$.

B. Magnification × 500.

C. Magnification × 1600.

Ultraviolet prints (above) and autoradiographs (below) of the corresponding ionophoretic runs of the nucleotide fractions of rat liver nuclei.

FIG. 4.—Short ionophoretic run. Component A shows up in the ultraviolet print in its characteristic crescentic shape. The nucleotides have not yet fully separated from each other.

FIG. 5.—Long ionophoretic run. The nucleotides are adequately separated and component H is visible between adenylic and guanylic acids.

*Cy.* = cytidylic acid. *Ad.* = adenylic acid.
*Gu.* = guanylic acid. *Ur.* = uridylic acid.

McIndoe and Davidson.

McIndoe and Davidson.

nucleotide fraction obtained from cytoplasmic materials by the Schmidt-Thannhauser (1945) separation procedure (Davidson and Smellie, 1952), although since the chemical nature of A, C and D is still unknown it is not possible to state whether the nuclear materials are identical with their cytoplasmic equivalents. Component B is essentially inorganic phosphate derived from " phosphoprotein " and corresponds to the fraction $P_2$ of Fig. 2.

Components A, C and D are organic phosphates, the presence of which is responsible, in part at least, for the difference between the " organic P " fraction in Table I and the RNAP by the orcinol reaction. The presence of such esters has already been suggested by Davidson and McIndoe (1949). It is clear that in nuclear tissue, as in whole tissue (Davidson, Frazer and Hutchison, 1951 ; Davidson and Smellie, 1952), the method of Schmidt and Thannhauser (1945) tends to give rather misleading results, since the RNA fraction contains, in addition to ribonucleotides, considerable amounts of non-ribonucleotide phosphate.

Incorporation studies with $^{32}P$ have revealed the rapid incorporation of the isotope into nuclear RNA and its slow incorporation into DNA in non-growing tissues.
Although nuclear RNA is much less abundant than cytoplasmic RNA it is more active, as has been shown by Marshak and Calvet (1949), Barnum and Huseby (1950), Jeener and Szafarz (1950) and Davidson, McIndoe and Smellie (1951) using $^{32}P$ and by Potter, Recknagel and Hurlbert (1951) using labelled orotic acid. The present studies show that this more rapid rate of incorporation of $^{32}P$ into nuclear RNA holds for all four nucleotides, which become labelled to practically the same extent.

The specific activities of components C and D are of the same order as those of the nucleotides 2 hours after administration of $^{32}P$, but that of the inorganic phosphate fraction is much higher (Table IV). This component corresponds to fraction $P_2$ in Table I and is derived mainly from " phosphoprotein," although it may contain traces of inorganic phosphate originally present in the tissue. Its presence is responsible for the fact that the activity of fraction " acid soluble 2 " is higher than that of " organic P." The high activity of fraction $P_2$ in whole tissue has already been commented on by Davidson, Frazer and Hutchison (1951).

The results of the experiments on fowls confirm the observation of Kelly and Jones (1951) and Kelly, Payne, White and Jones (1951) in rats that the presence of a rapidly growing tumour in the animal body leads to an increased incorporation of $^{32}P$ into the liver DNAP. This effect is still unexplained and obviously requires much further examination, since it is associated in our experiments with the rather high specific activity of normal fowl liver DNA as compared with rat or rabbit liver DNA. The high activity for the DNA of the GRCH 15 tumour is in agreement with the results of other workers for rapidly growing tissues (Brues, Tracey and Cohn, 1944 ; Davidson and Raymond, 1948).

The results presented in Table II are of some interest in relation to the claims of Vendrely and Vendrely (1948, 1949) and of Mirsky and Ris (1949) that the DNA content of the cell nucleus is apparently constant for the cells of the different somatic tissues of any one species although there may be wide variations between one species and another. The implications of these observations have been discussed by Davidson and Leslie (1950*a*, *b*). The figures recorded in Table II

for the mean DNA content of the nucleus are in accord with those of other workers (for table see Davidson and Leslie, 1950*b*), and the results obtained by three different methods of estimation are of the same order, although the figures for the diphenylamine method are in general slightly lower than those obtained by phosphorus estimations or ultraviolet absorption measurements.

The mean DNA content of the rat liver nucleus (9·65 pg.), which is higher than for other rat tissues (about 6·5 pg. per nucleus) (Davidson, Heagy and Thomson, 1952, unpublished results), is apparently due to the occurrence of polyploidy (Swift, 1950). As might be expected, no loss of DNA from the rat liver nucleus occurs on fasting, although the RNA content falls. Too much stress however should not be laid on this latter observation, since the presence of minute amounts of cytoplasmic contamination could significantly alter the RNA content of the nucleus. It has already been shown (Davidson, 1947) that fasting causes a marked fall in the total RNA content of the rat liver, while the total DNA content is unchanged. This would of course be expected if the DNA content of the individual nuclei and the number of nuclei per liver were unaltered.

In the GRCH 15 fowl tumour the DNA content of the nucleus is approximately double that in the fowl erythrocyte or liver cell. The significance of this observation is as yet obscure, but a similar high value for tumour cells has been recorded by Klein (1951) for several mouse tumours. On the other hand normal values for the DNA content of tumour cell nuclei have been recorded for rat hepatoma by Mark and Ris (1949), Cunningham, Griffin and Luck (1950), Price, Miller, Miller and Weber (1950), and by Davidson, Heagy and Thomson (1952, unpublished results), for mouse lymphoma by Klein (1951), and for leukemic cells in man by Davidson, Leslie and White (1951).

SUMMARY.

1. The composition of cell nuclei isolated in bulk, from calf thymus, rabbit, rat and fowl liver, fowl erythrocytes and the GRCH 15 tumour is expressed in terms of dry weight and in terms of statistical amount per single nucleus.

2. The cell nucleus contains, in addition to DNA, appreciable amounts of RNA. By means of ionophoresis on paper it has been shown that nuclear RNA contains cytidylic acid, adenylic acid, guanylic acid and uridylic acid, but the ribonucleotide fraction prepared from nuclei by the Schmidt-Thannhauser technique contains in addition to these four nucleotides several other phosphate esters and a small amount of inorganic phosphate derived from "phosphoprotein."

3. The incorporation of $^{32}P$ into the nuclear components has been studied. The nuclear RNA incorporates the isotope much more readily than does cytoplasmic RNA. The rate of incorporation of $^{32}P$ into DNA is low in resting tissues.

Our grateful thanks are due to Mr. D. R. S. Cameron and Miss R. Pevie for their skilful technical assistance. We also acknowledge with thanks that the expenses of this work were defrayed in part by grants from the British Empire Cancer Campaign, the Medical Research Council and the Rankine Fund of the University of Glasgow.

### REFERENCES.

Allen, R. J. L.—(1940) *Biochem. J.*, **34**, 858.
Barnum, C. P., and Huseby, R. A.—(1950) *Arch. Biochem.*, **29**, 7.
Beale, R. N., Harris, R. J. C., and Roe, E. M. F.—(1950) *J. chem. Soc.*, 1397.
Bergstrand, A., Eliasson, N. A., Hammarsten, E., Norberg, B., Reichard, P., and Ubisch, H.—(1948) *Cold Spring Harbor Symp. Quant. Biol.*, **13**, 22.
Brachet, J.—(1942) *Acta biol. Belg.*, **1**, 13.
Brues, A. M., Tracey, M. M., and Cohn, W. E.—(1944) *J. biol. Chem.*, **155**, 619.
Caspersson, T., and Schultz, J.—(1940) *Proc. nat. Acad. Sci.*, **26**, 507.
Chargaff, E.—(1950) *Experentia*, **6**, 201.
Consden, R., Gordon, A. H., and Martin, A. J. P.—(1947) *Biochem. J.*, **40**, 33.
Cunningham, L., Griffen, A. C., and Luck, J. M.—(1950) *Cancer Res.*, **10**, 211.
Davidson, J. N.—(1947) *Cold Spring Harbor Symp. Quant. Biol.*, **12**, 50
*Idem*, Frazer, S. C., and Hutchison, W. C.—(1951) *Biochem. J.*, **49**, 311.
*Idem*, Gardner, M., Hutchison, W. C., McIndoe, W. M., Raymond, W. H. A., and Shaw, J. F.—(1949) *Ibid.*, **44**, Proc. xx.
*Idem* and Leslie, I.—(1950*a*) *Nature*, **165**, 49.—(1950*b*) *Cancer Res.*, **10**, 587.
*Iidem*, Smellie, R. M. S., and Thompson, R. Y.—(1950) *Biochem. J.*, **46**, Proc. xl.
*Idem*, Leslie, I., and White, J. C.—(1951) *J. Path. Bact.*, **63**, 471.
*Idem* and McIndoe, W. M.—(1949) *Biochem. J.*, **45**, Proc. xvi.
*Iidem* and Smellie, R. M. S.—(1951) *Ibid.*, **49**, Proc. xxxvi.
*Idem* and Raymond, W.—(1948) *Ibid.*, **42**, Proc. xiv.
*Idem* and Smellie, R. M. S.—(1952) *Ibid.* (in the Press).
*Idem* and Waymouth, C.—(1944*a*) *Ibid.*, **38**, 39.—(1944*b*) *Ibid.*, **38**, 379.—(1946) *J. Physiol.*, **105**, 191.
Dische, Z.—(1930) *Mikrochemie*, **8**, 4.
Dounce, A. L., and Lan, T. H.—(1943) *Science*, **97**, 584.
*Idem*, Tishkoff, G. H., Barnett, S. R., and Frier, R. M.—(1950) *J. gen. Physiol.*, **33**, 629.
Elson, D., and Chargaff, E.—(1951) *Fed. Proc.*, **10**, 180.
Euler, H., and Hahn, L.—(1946) *Särtyrck ur. Svensk Kemisk Tidskrift*, **58**, 251.
Holiday, E. R., and Johnson, E. A.—(1949) *Nature*, **163**, 216.
Jeener, R., and Szafarz, D.—(1950) *Arch. Biochem.*, **26**, 54.
Kelly, L. S., and Jones, H. B.—(1951) *Science*, **111**, 333.
*Idem*, Payne, A. H., White, M. R., and Jones, H. B.—(1951) *Cancer Res.*, **11**, 694.
Klein, G.—(1951) *Exp. Cell Res.*, **2**, 518.
Lagerstedt, S.—(1948) *Acta anat., Basel*, Suppl. 9.
Mark, D. D., and Ris, H.—(1949) *Proc. Soc. exp. Biol., N.Y.*, **71**, 727.
Markham, R., and Smith, J. D.—(1949) *Biochem. J.*, **45**, 294.
Marshak, A.—(1951) *J. biol. Chem.*, **189**, 607.
*Idem* and Calvet, F.—(1949) *J. cell comp. Physiol.*, **34**, 451.
*Idem* and Vogel, H. J.—(1950) *Fed. Proc.*, **9**, 85.
Mathison, G. C.—(1909) *Biochem. J.*, **4**, 233.
Mejbaum, W.—(1939) *Z. physiol. Chem.*, **2**, 258, 117.
Mirsky, A., and Pollister, A. W.—(1946) *J. gen. Physiol.*, **30**, 117.
*Idem* and Ris, H.—(1947) *Ibid.*, **31**, 1.—(1949) *Nature*, **163**, 666.
Nash, C. W.—(1949) *Canad. pharm. J.*, **82**, 251.
Peacock, P. R.—(1933) *J. Path. Bact.*, **36**, 141.
Pollister, A. W., and Leuchtenberger, C.—(1949*a*) *Nature*, **163**, 360.—(1949*b*) *Proc. nat. Acad. Sci.*, **35**, 66.
Potter, V. R., Recknagel, R. O., and Hurlbert, R. B.—(1951) *Fed. Proc.*, **10**, 646.
Price, J. M., Miller, E. C., Miller, J. A., and Weber, G. M.—(1950) *Cancer Res.*, **10**, 18.
Ris, H., and Mirsky, A.—(1949) *J. gen. Physiol.*, **33**, 125.

SCHMIDT, G., AND THANNHAUSER, A. J.—(1945) *J. biol. Chem.*, **161**, 83.
SCHNEIDER, W. C.—(1945) *Ibid.*, **161**, 293.—(1946) *Ibid.*, **164**, 747.
SMELLIE, R. M. S., AND DAVIDSON, J. N.—(1951) *Biochem. J.*, **49**, Proc. xv.
STEDMAN, E., AND STEDMAN, E.—(1943) *Nature*, **152**, 267.—(1947) *Cold Spring Harbor Symp. Quant. Biol.*, **12**, 224.—(1951) *Phil. Trans. Roy. Soc., Lond.*, **235**B, 565.
SWIFT, H.—(1950) *Physiol. Zoöl.*, **23**, 169.
VENDRELY, R., AND VENDRELY, C.—(1948) *Experentia*, **4**, 434.—(1949) *Ibid.*, **5**, 327.
VILLELA, G. G.—(1949) *Nature*, **164**, 667.
WYATT, G. R.—(1951) *Biochem. J.*, **48**, 584.


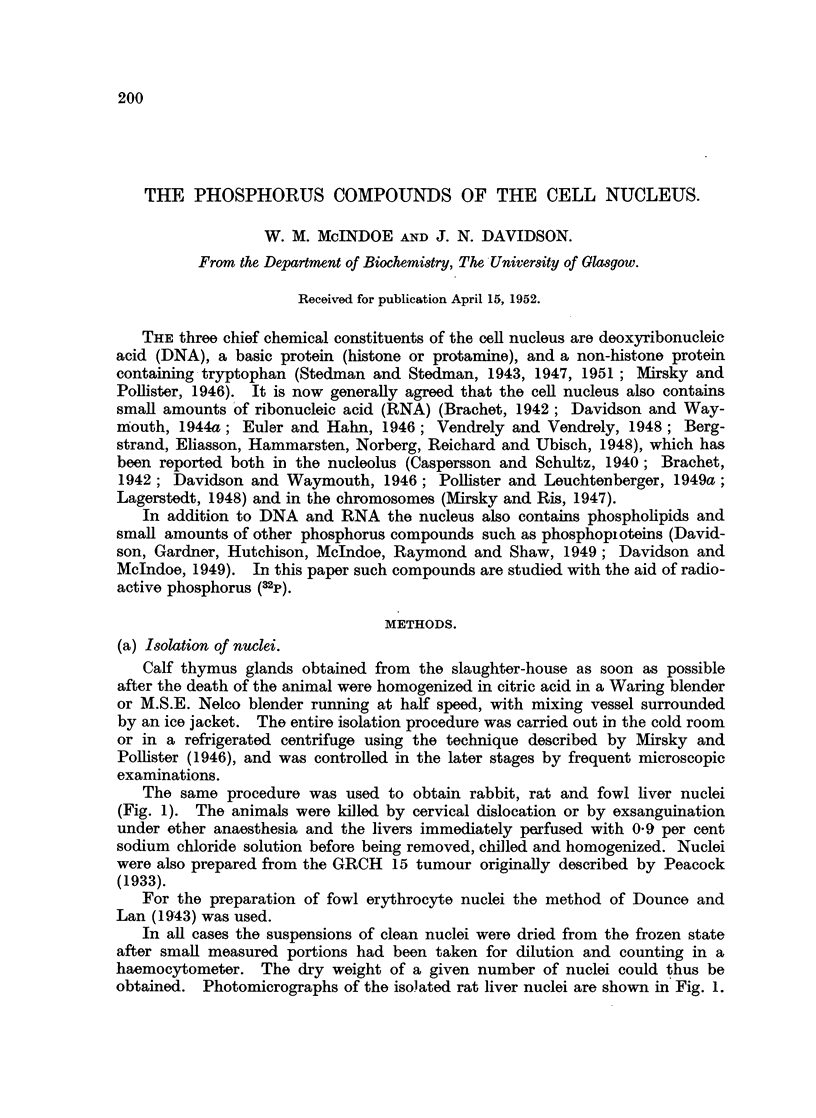

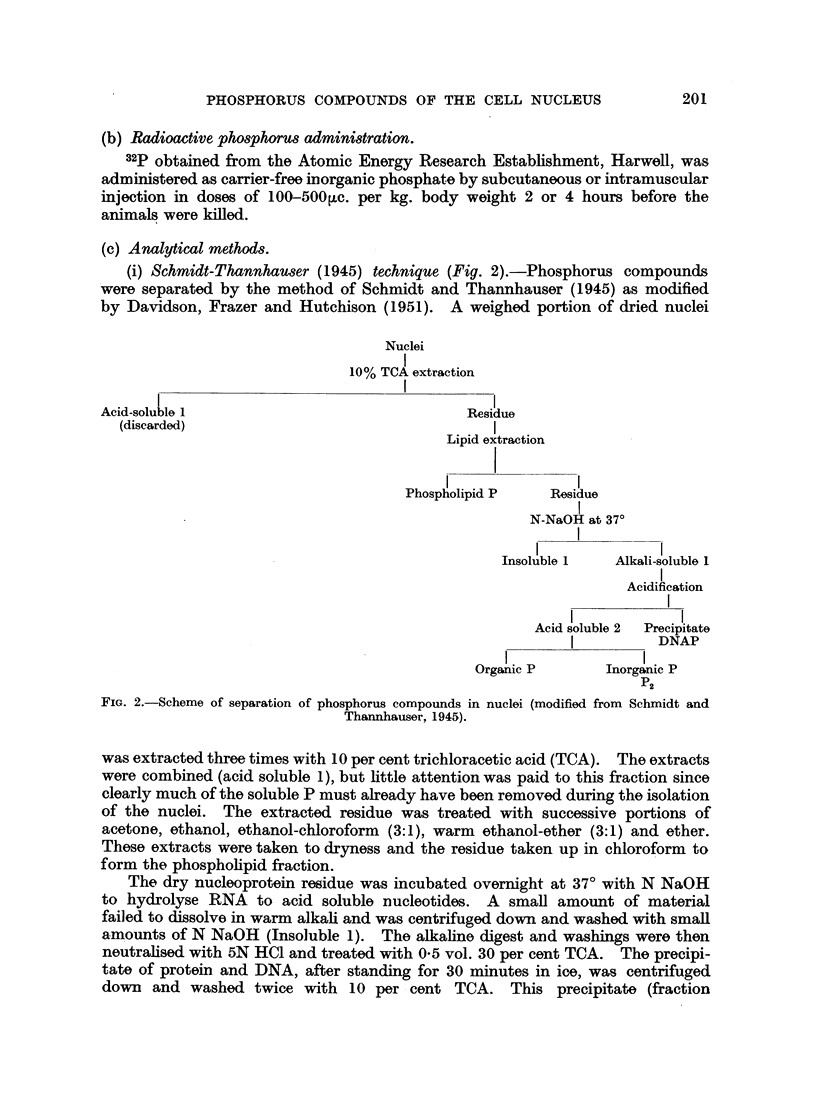

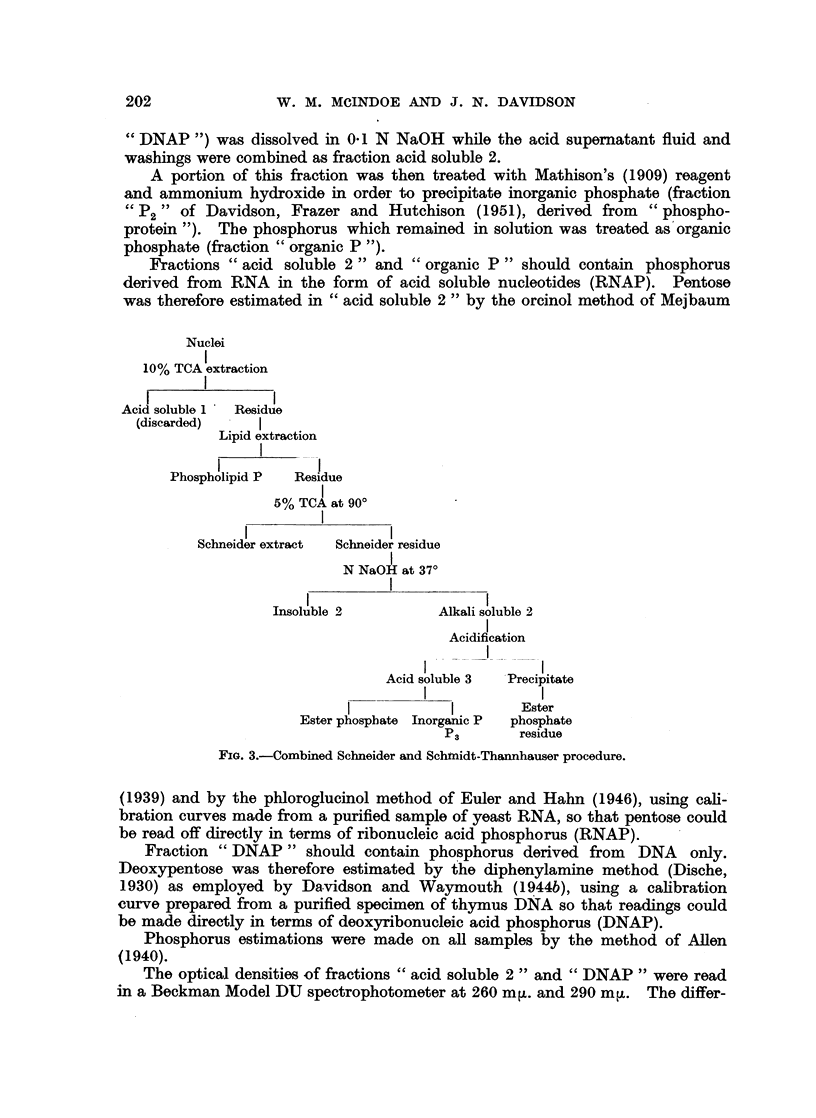

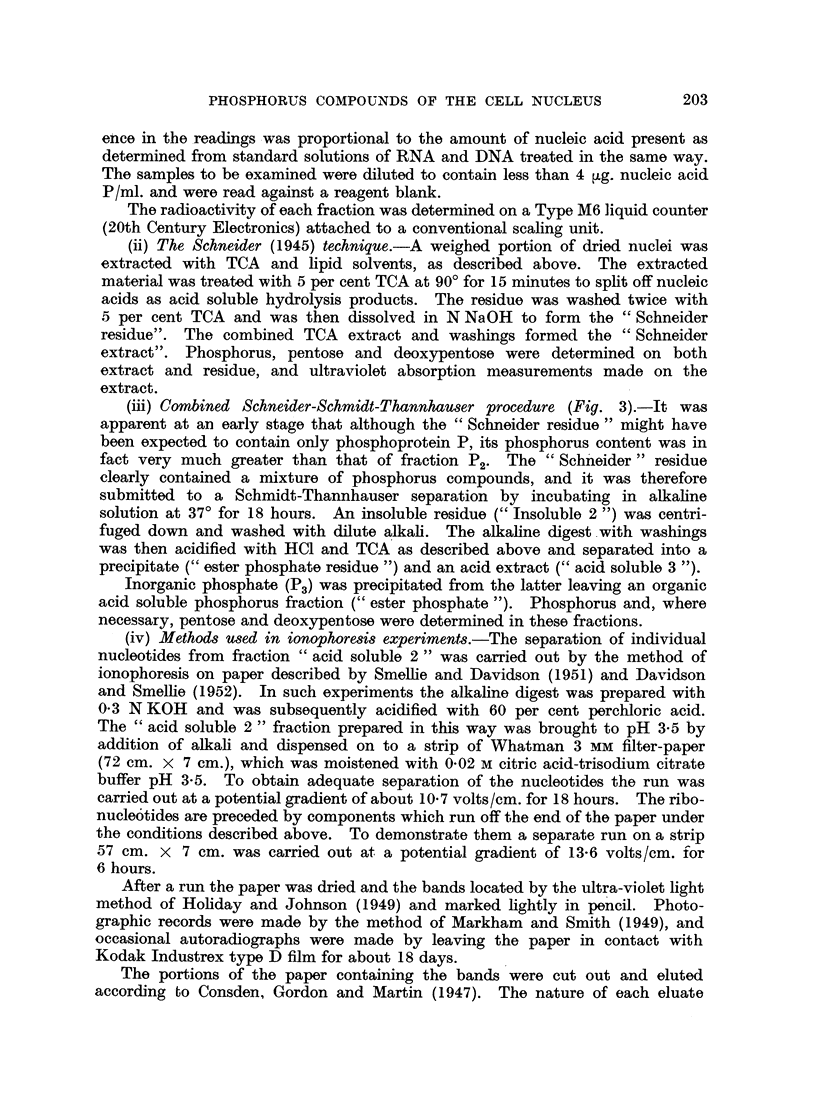

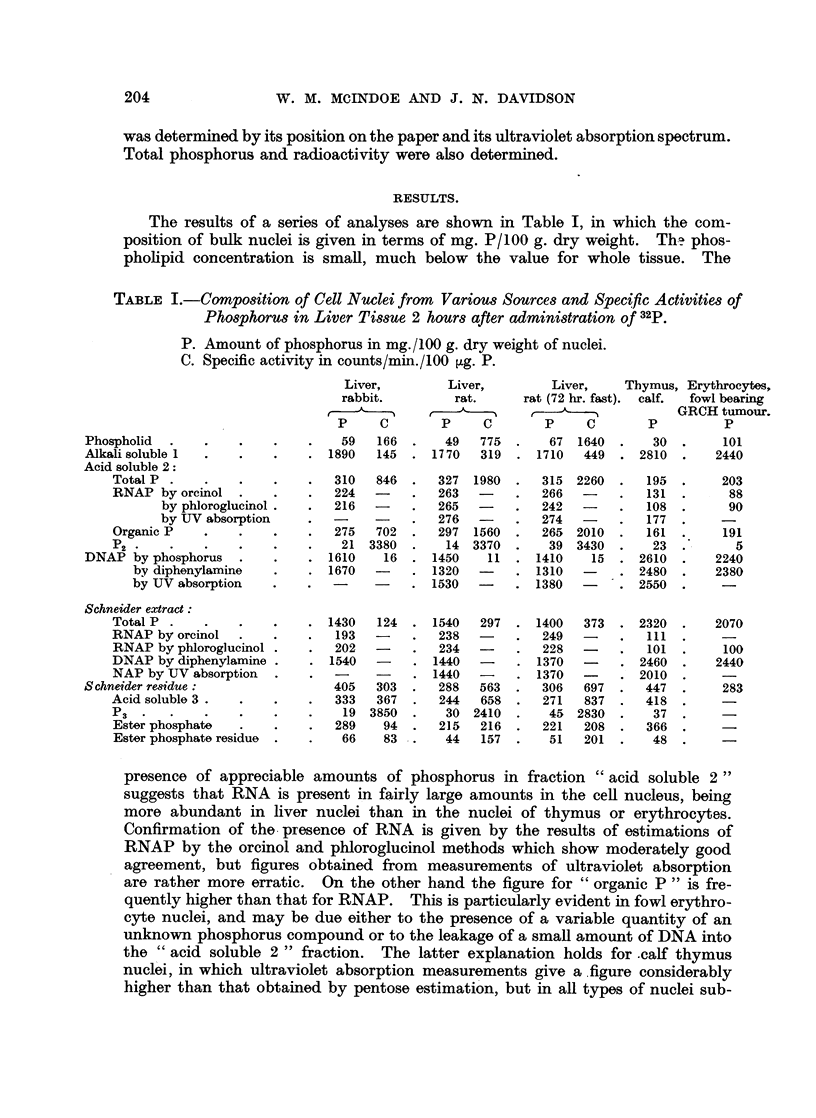

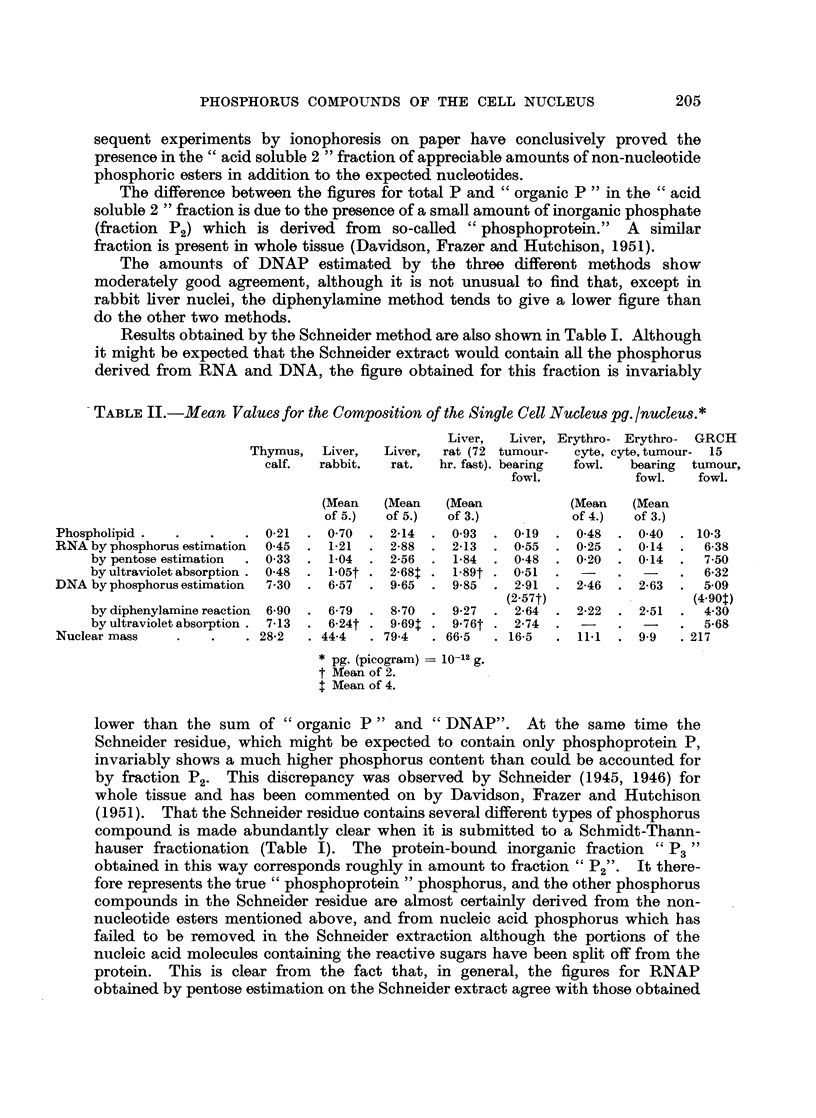

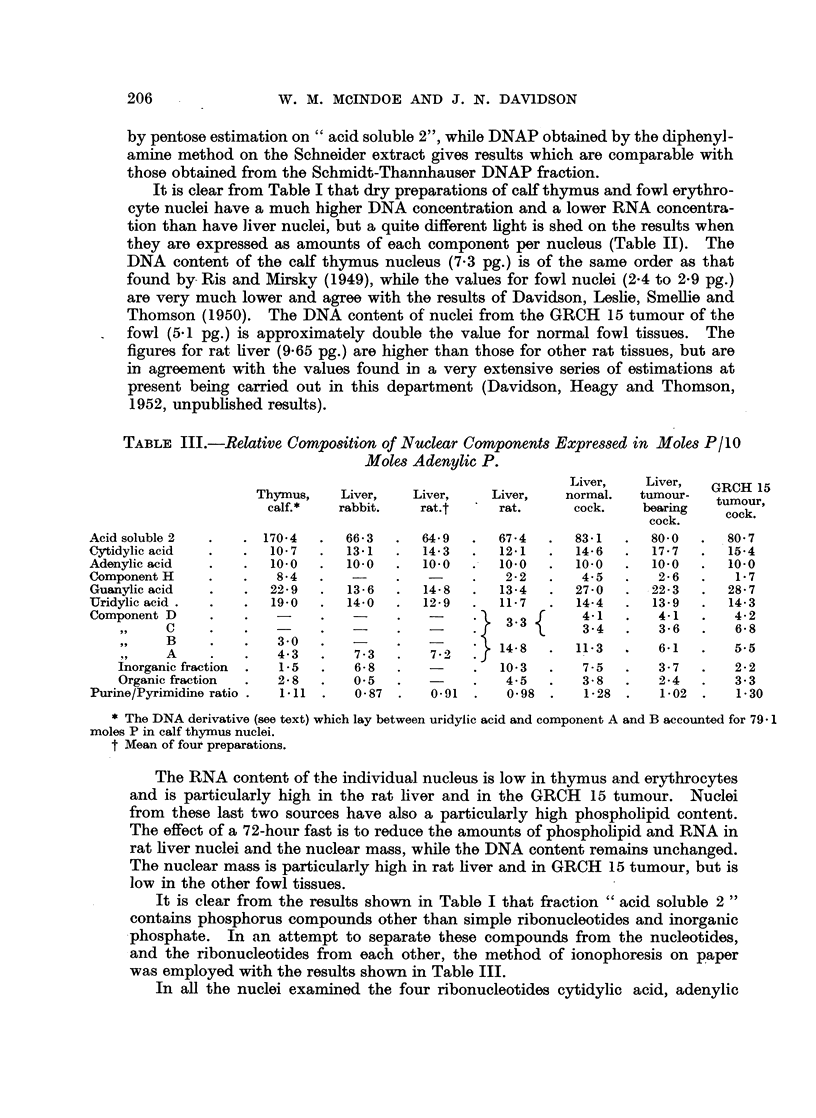

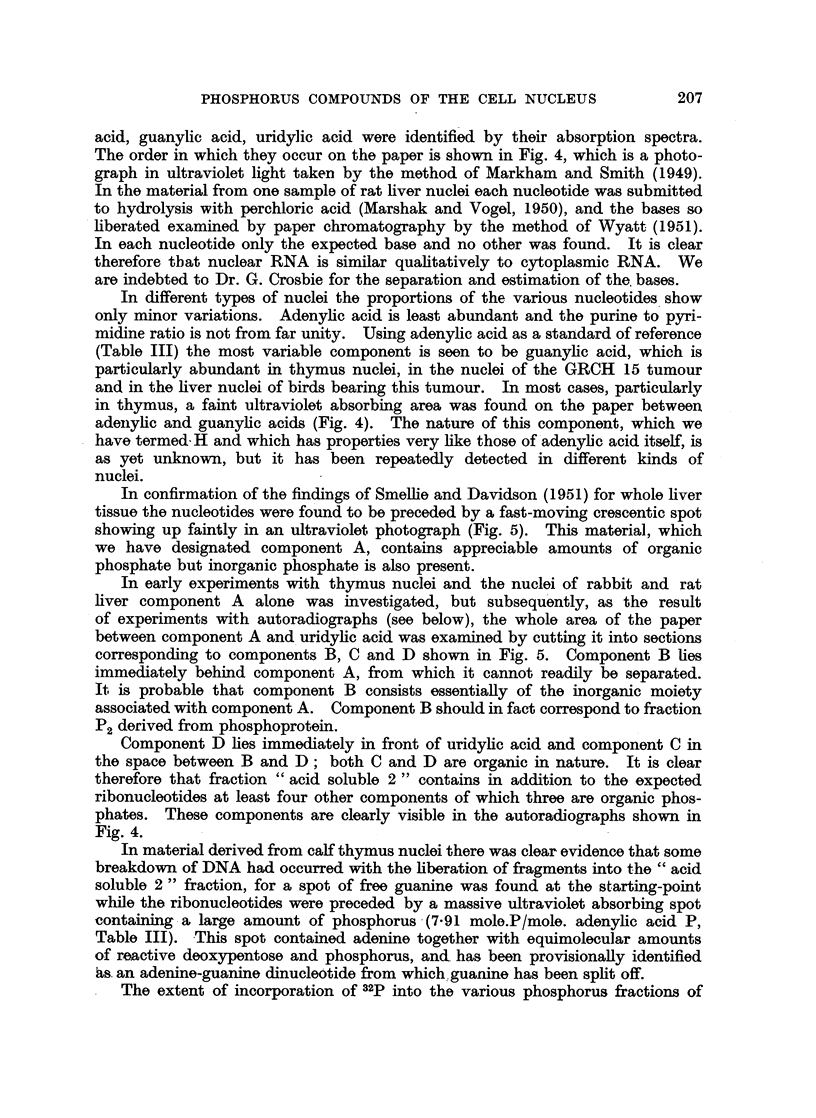

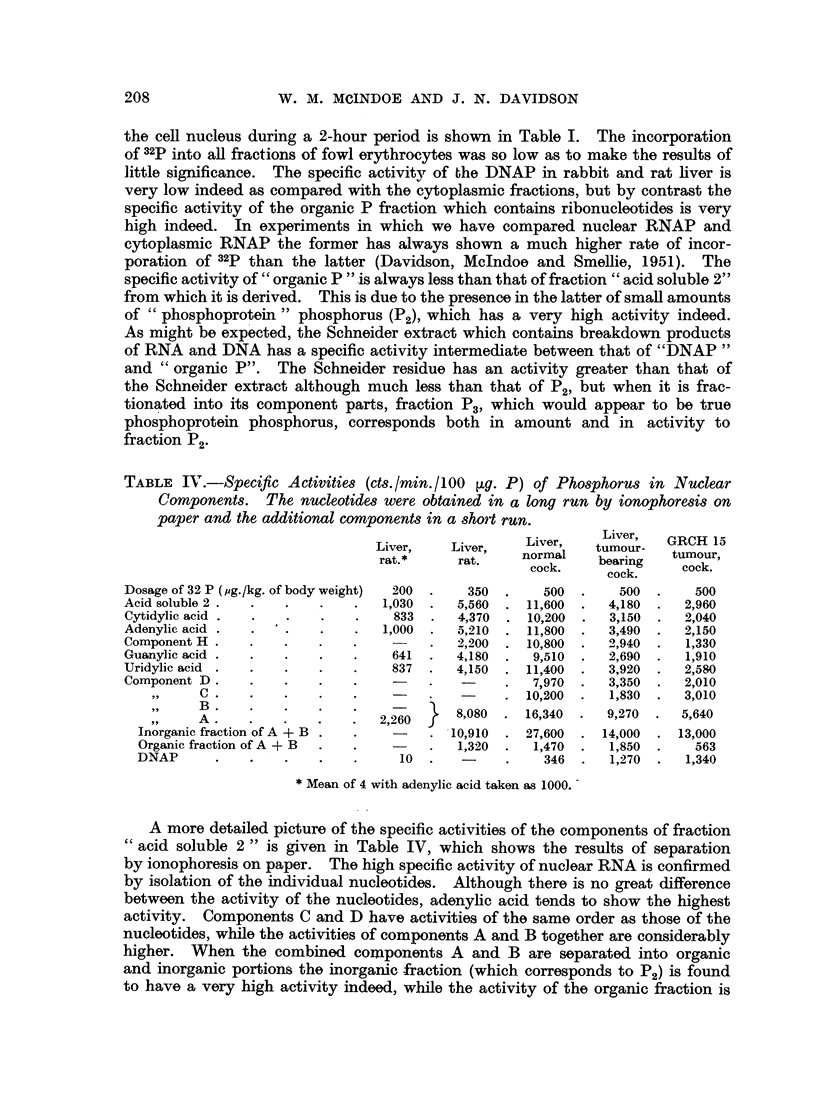

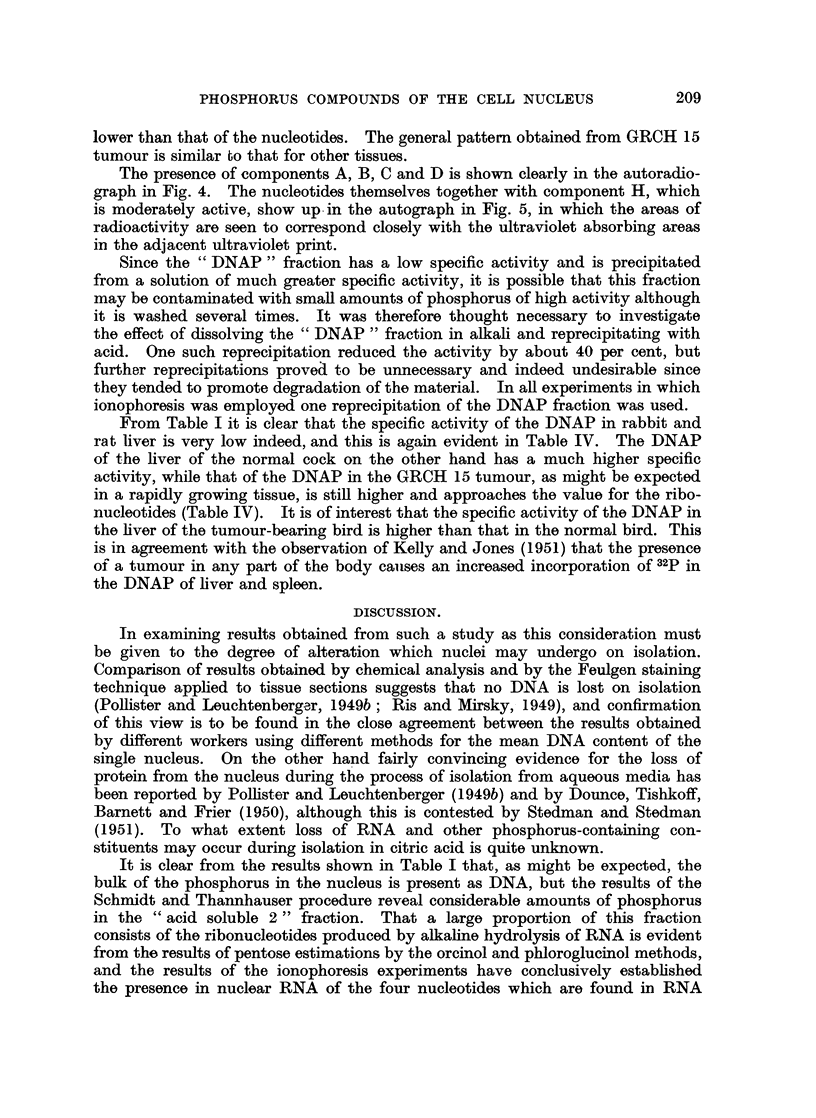

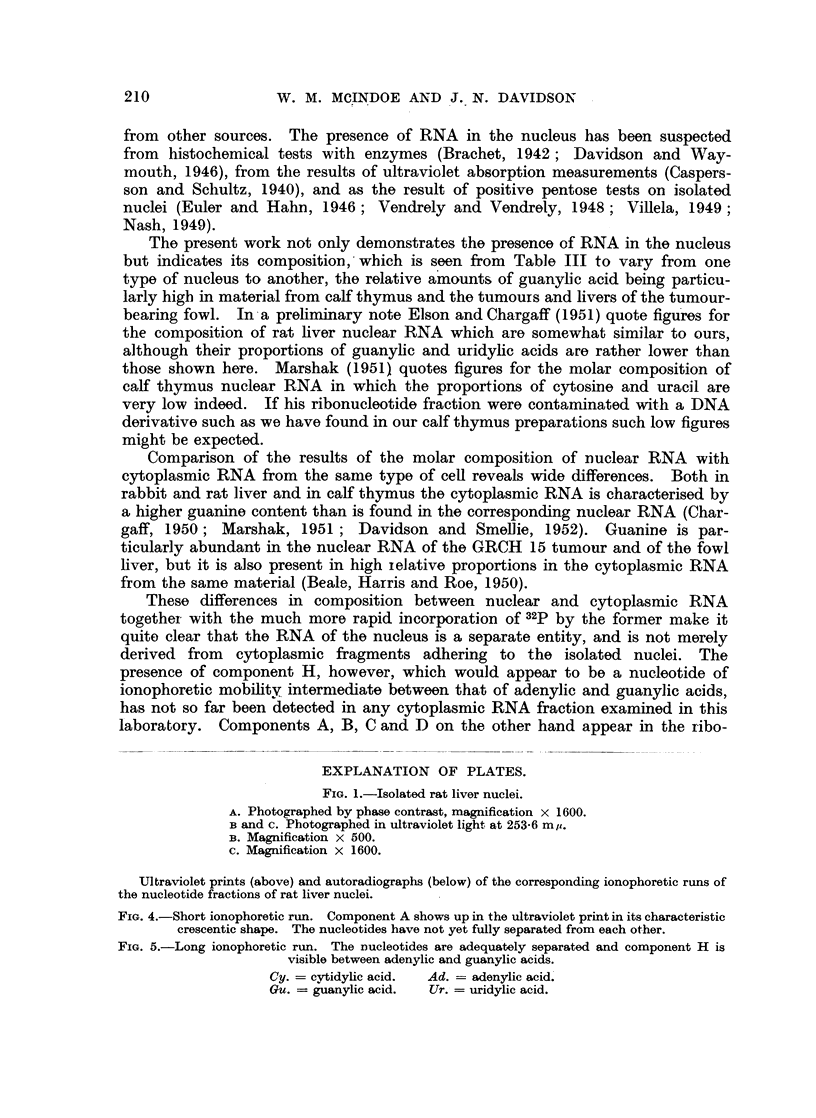

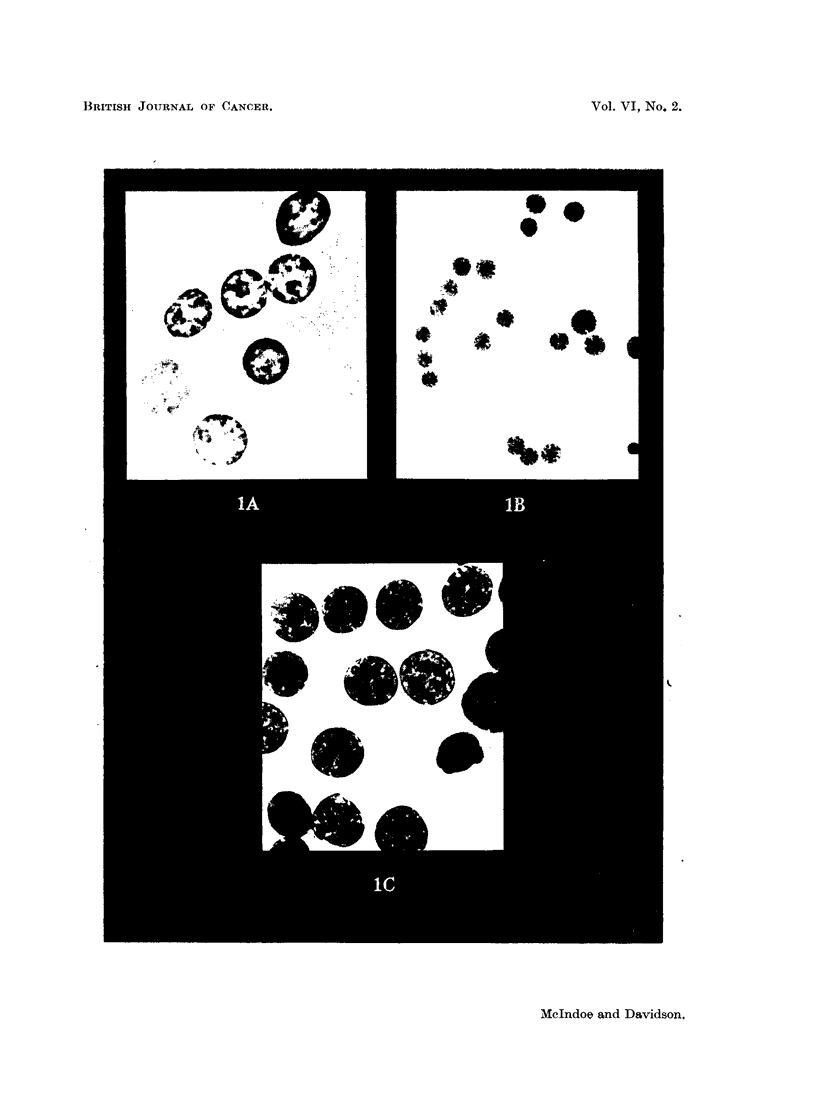

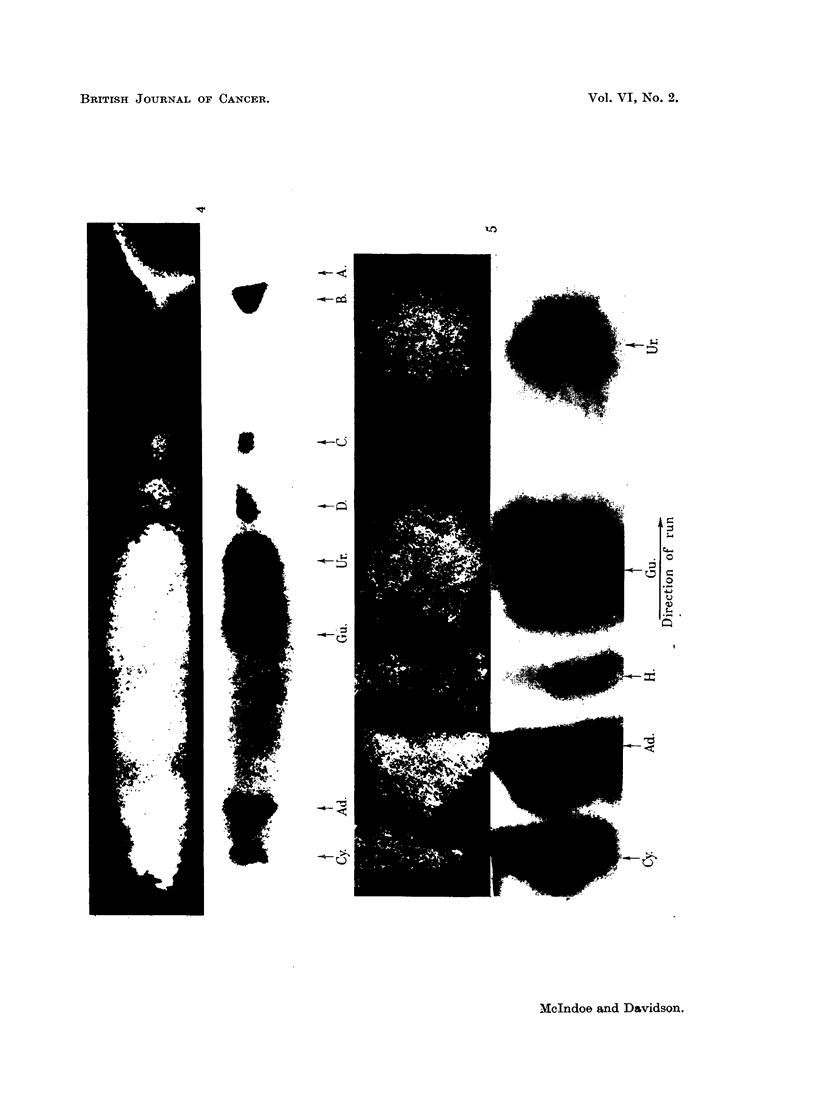

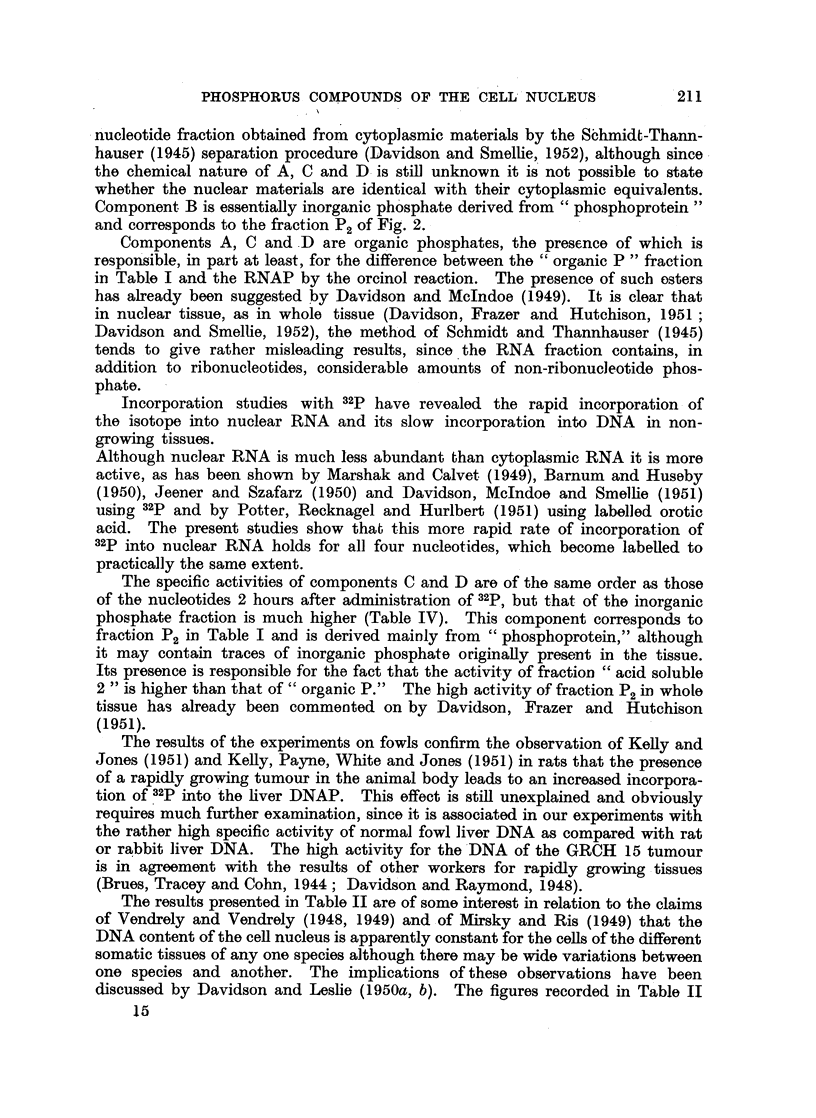

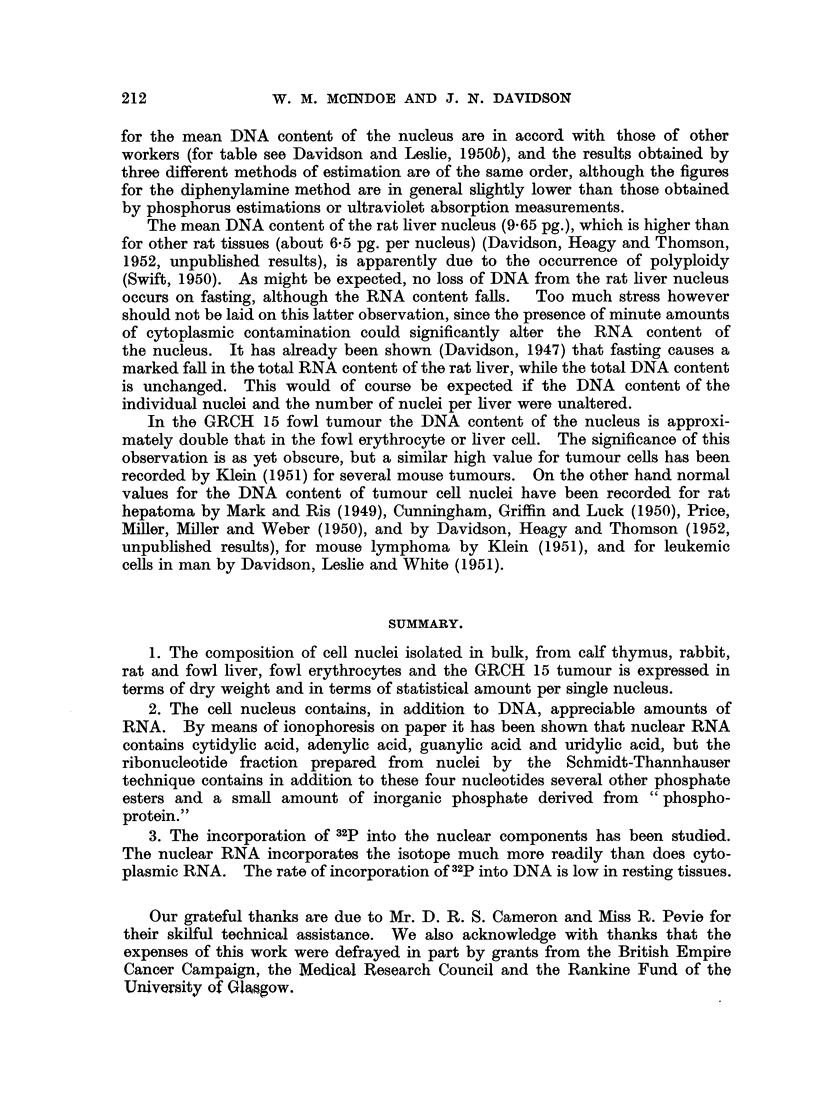

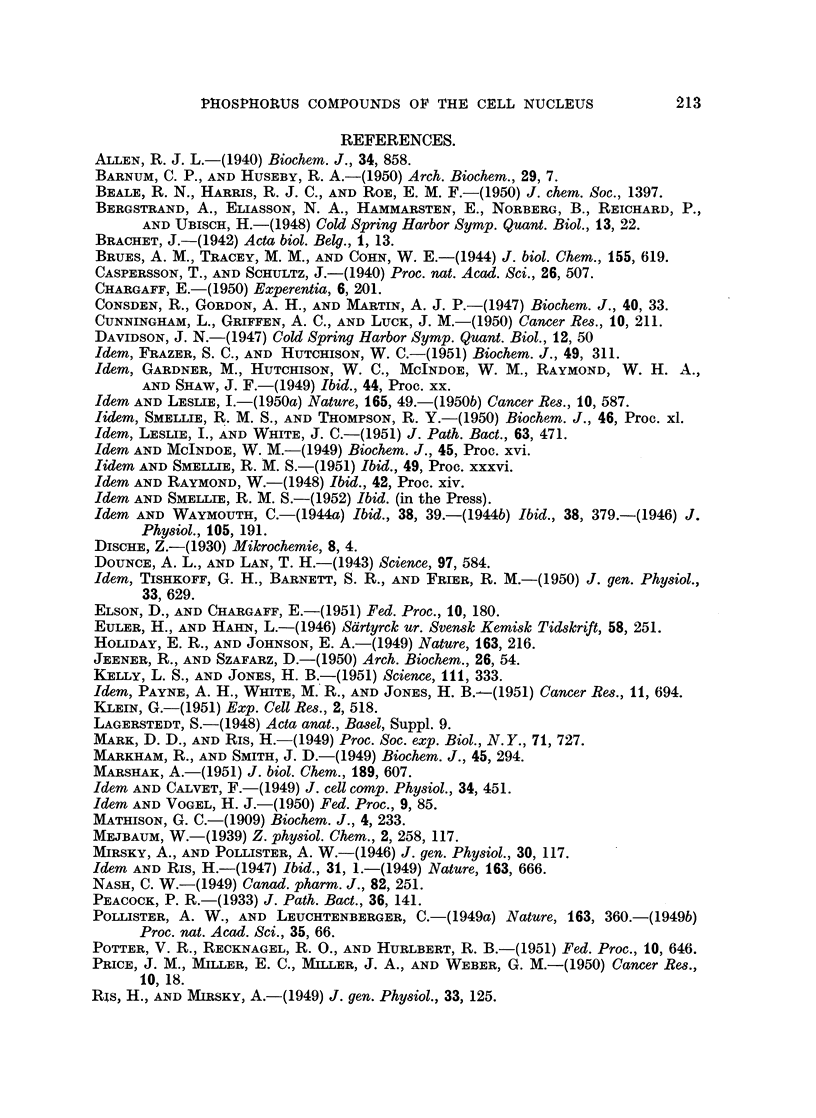

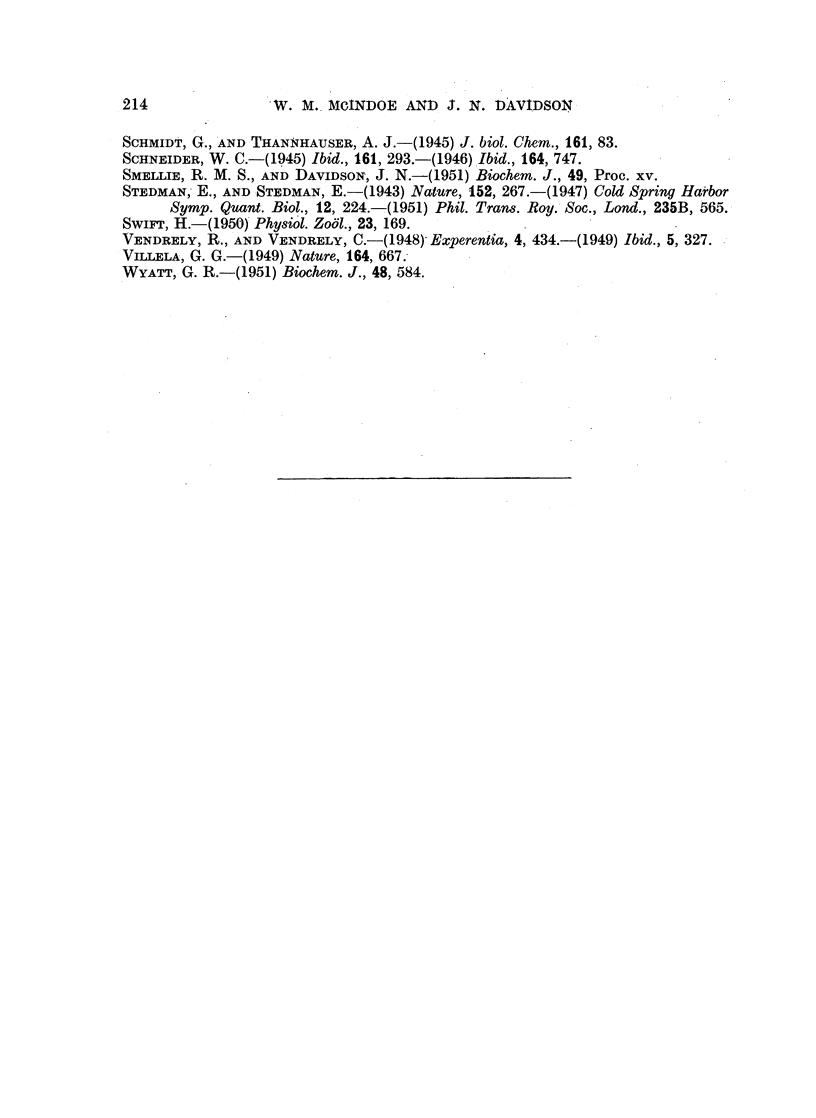


## References

[OCR_00890] Allen R. J. (1940). The estimation of phosphorus.. Biochem J.

[OCR_00903] CHARGAFF E. (1950). Chemical specificity of nucleic acids and mechanism of their enzymatic degradation.. Experientia.

[OCR_00907] DAVIDSON J. N., FRAZER S. C., HUTCHISON W. C. (1951). Phosphorus compounds in the cell. I. Protein-bound phosphorus fractions studied with the aid of radioactive phosphorus.. Biochem J.

[OCR_00917] DAVIDSON J. N., LESLIE I., WHITE J. C. (1951). Quantitative studies on the content of nucleic acids in normal and leukaemic cells, from blood and bone marrow.. J Pathol Bacteriol.

[OCR_00931] DOUNCE A. L., TISHKOFF G. H., BARNETT S. R., FREER R. M. (1950). Free amino acids and nucleic acid content of cell nuclei isolated by a modification of Behrens' technique.. J Gen Physiol.

[OCR_00925] Davidson J. N., Waymouth C. (1946). The nucleoproteins of the liver cell demonstrated by ultra-violet microscopy.. J Physiol.

[OCR_00929] Dounce A. L., Lan T. H. (1943). ISOLATION AND PROPERTIES OF CHICKEN ERYTHROCYTE NUCLEI.. Science.

[OCR_00943] KELLY L. S., PAYNE A. H., WHITE M. R., JONES H. B. (1951). The effect of neoplasia or pregnancy on the tissue desoxypentosenucleic acid.. Cancer Res.

[OCR_00948] MARKHAM R., SMITH J. D. (1949). Chromatographic studies of nucleic acids; a technique for the identification and estimation of purine and pyrimidine bases, nucleosides and related substances.. Biochem J.

[OCR_00952] MARSHAK A., CALVET F. (1949). Specific activity of P32 in cell constituents of rabbit liver.. J Cell Physiol.

[OCR_00953] Mathison G. C. (1909). The Estimation of Phosphorus in Urine.. Biochem J.

[OCR_00966] POTTER V. R., RECKNAGEL R. O., HURLBERT R. B. (1951). Intracellular enzyme distribution; interpretations and significance.. Fed Proc.

[OCR_00973] RIS H., MIRSKY A. E. (1949). Quantitative cytochemical determination of desoxyribonucleic acid with the Feulgen nucleal reaction.. J Gen Physiol.

[OCR_00983] SWIFT H. H. (1950). The desoxyribose nucleic acid content of animal nuclei.. Physiol Zool.

[OCR_00988] WYATT G. R. (1951). The purine and pyrimidine composition of deoxypentose nucleic acids.. Biochem J.

